# Ketogenic Diet: A Dietary Intervention via Gut Microbiome Modulation for the Treatment of Neurological and Nutritional Disorders (a Narrative Review)

**DOI:** 10.3390/nu14173566

**Published:** 2022-08-30

**Authors:** Jun-Ming Lim, Vengadesh Letchumanan, Loh Teng-Hern Tan, Kar-Wai Hong, Sunny-Hei Wong, Nurul-Syakima Ab Mutalib, Learn-Han Lee, Jodi Woan-Fei Law

**Affiliations:** 1 Novel Bacteria and Drug Discovery Research Group (NBDD), Microbiome and Bioresource Research Strength (MBRS), Jeffrey Cheah School of Medicine and Health Sciences, Monash University Malaysia, Bandar Sunway 47500, Malaysia; 2 Clinical School Johor Bahru, Jeffrey Cheah School of Medicine and Health Sciences, Monash University Malaysia, Johor Bahru 80100, Malaysia; 3 Lee Kong Chian School of Medicine, Nanyang Technological University, Singapore 308232, Singapore; 4 State Key Laboratory of Digestive Disease, Department of Medicine and Therapeutics, The Chinese University of Hong Kong, Hong Kong SAR, China; 5 UKM Medical Molecular Biology Institute (UMBI), Universiti Kebangsaan Malaysia, Kuala Lumpur 56000, Malaysia; 6 Faculty of Health Sciences, Universiti Kebangsaan Malaysia, Kuala Lumpur 50300, Malaysia

**Keywords:** dietary therapies, nutrition, diet, gut microbiota, intestinal microbes, microbiome

## Abstract

The ketogenic diet (KD) has been important in treating epilepsy since the 1920s. The benefits of KD further expanded to other neurological diseases, including Alzheimer’s diseases, autism spectrum disorder, and nutritional disorder (obesity). Although the therapeutic efficacy of KD has been generally accepted, there is limited knowledge about its underlying mechanism of action, particularly its effect on our gut microbiome. Gut dysbiosis has been proposed to be involved in those diseases, and KD can promote gut microbiota remodeling that may assist in recovery. This review explores the therapeutic applications of KD, the roles of the gut microbiome in neurological diseases and obesity, as well as the effect of KD on the gut microbiome. The present information suggests that KD has significant roles in altering the gut microbiome to improve disease symptoms, mainly by incrementing *Bacteroidetes* to *Firmicutes* (B/F) ratio and reducing *Proteobacteria* in certain cases. However, current gaps call for continued research to understand better the gut microbiota profile altered by KD.

## 1. Introduction

The ketogenic diet (KD) was first introduced by Dr. Russel Wilder in 1921 and is characterized by a high fat, moderate protein, and a low carbohydrate diet [1]. The classic KD is based on a gold standard ratio of fats to a combination of proteins and carbohydrates in 4:1 [1,2]. In this dietary plan, fat contributes 90% of calories, while protein and carbohydrate give 6% and 4% calories, respectively [3]. This dietary approach was extensively used for treating epilepsy before antiepileptic agents were introduced in 1938 [1]. Eventually, various forms of less restrictive ketogenic diets (KDs), including medium-chain triglyceride diet, modified Atkins diet, and low glycaemic index treatment, with different fat to protein to carbohydrate ratios, have been introduced to accommodate the diverse lifestyles and for improved tolerance [4].

From physiology and biochemistry aspects, the KD approach involves the deprivation of carbohydrates that mimics starvation, reducing the glycolysis process for energy generation. The body will undergo glycogenolysis by catabolizing glycogen from muscle and liver into glucose as alternate energy sources. In addition, gluconeogenesis is an alternative pathway that utilizes amino acids from protein while fatty acids and glycerol from fat. Lipolysis generates ketone (acetoacetate, acetone, and beta-hydroxybutyrate acid) through β oxidation and ketogenesis [5]. Ketone, the primary energy source in the ketosis state, can be used by the heart, renal, and muscles. Additionally, ketone bodies crossing the blood–brain barrier are recognized as direct anticonvulsants [6]. Nevertheless, emerging evidence revealed that the process of ketosis producing ketone might not be the sole mechanism of action in providing therapeutic effects. Other proposed therapeutic mechanisms of KD include increment in norepinephrine neurotransmitter release, enhancement of gamma-aminobutyric acid level, reduction in the production of reactive oxygen species, induction of the expression of neuronal uncoupling protein, upregulation of energy metabolism gene, mitochondrial biogenesis, hyperpolarization of neurons that could reduce neuronal excitability, and upregulation of K_ATP_ channels [7].

Meanwhile, KD significantly impacts gut microbiota, leading to changes in metabolite levels and mediating therapeutic effects [8]. The gut microbiome is vital in affecting health and diseases [9,10,11,12]. Diet or diet-based therapies (e.g., KD and low FODMAP diet) are among the most vital modifiable factors regulating the gut microbiome composition [13]. Our enteric nervous system acts as our second brain, which depends on serotonin neurotransmitters similar to our central nervous system. The brain–gut axis communicates bi-directionally, in which the intestinal microbiota is likely to influence neurotransmission in the central nervous system and vice versa [14]. Since the therapeutic dietary plan can affect microbial composition and diversity, targeting the gut microbiome could potentially help treat diseases and enhance overall health. Recently, the advantages of the KD have been expanded from epilepsy to other neurodegenerative conditions such as Alzheimer’s disease and autism spectrum disorder, and nutritional disorders such as obesity [15,16]. Additionally, increasing evidence suggests that gut microbiome dysbiosis is associated with the pathophysiology of epilepsy, Alzheimer’s disease, autism spectrum disorder, and obesity [17]. Hence, it is anticipated that KD could render beneficial effects by improving and restoring the gut microbiome to a premorbid state. The present review aims to explore the recent therapeutic applications of the KD. This review will also discuss the effects of KD on the modulation of gut microbiome composition and the potential mechanisms that contributed to the therapeutic outcomes.

## 2. Therapeutic Application of Ketogenic Diet in Different Diseases and Its Association with Gut Microbiome Modulation

### 2.1. Epilepsy

The effectiveness of KD in treating epilepsy in children has been demonstrated in a randomized controlled trial conducted by Neal et al. (2008) [18]. In the trial, 54 epileptic children allocated to the KD group showed a clinically significant reduction by 75% in the mean percentage of baseline seizures compared to the 49 epileptic children in the control group three months after diet intervention. KD has also proven to be effective in adults; a study involving 23 individuals with epilepsy demonstrated 39% of them achieved ≥50% reduced seizure frequency, 22% of them showed <50% reduction or inconsistently ≥50% decrease in seizure frequency [19].

Meanwhile, the hypothesis regarding the association between the gut microbiome and epilepsy started in 1916 when Dr. Charles Reed linked epilepsy between constipation and *Bacillus epilepticus* [20]. Researchers began to study the patients’ gut microbiome and its potential association with the pathogenesis of epilepsy, given the availability of advanced genomic technologies. In one of the latest studies, Safak et al. discovered a significantly higher proportion of *Proteobacteria* (*Campylobacter*, *Delftia*, *Haemophilus*, *Lautropia* and *Neisseria*) and a lower proportion of *Firmicutes* (synonym *Bacillota*), *Bacteroidetes* (synonym *Bacteroidota*), and *Actinobacteria* (synonym *Actinomycetota*) in adult participants with epilepsy as compared to that of healthy participants. Interestingly, they also discovered that *Fusobacteria* (*Leptotrichia*, *Fusobacterium*) was absent in the control group and only detected in the epileptic group [21]. Likewise, Xie and the collaborators also found a higher proportion of *Proteobacteria* and a lower proportion of *Bacteroidetes* and *Actinobacteria* in infants with refractory epilepsy. However, the predominant bacterial population in epileptic infants was the *Firmicutes*. Moreover, epileptic infants possessed lower gut microbiota diversity [22] (Table 1). Peng et al. also revealed a relatively greater abundance of *Firmicutes* and *Verrucomicrobia* and a lower level of *Bacteroidetes* in the drug-resistant epilepsy group than that of drug-sensitive plus healthy participants. Notably, participants with drug-resistant epilepsy had an increased abundance of rare gut microbiome compositions such as *Atopobium*, *Dorea*, *Delftia*, *Coprobacillus*, *Clostridium* XVIII, *Saccharibacteria*, and others. The drug-sensitive epilepsy participants and healthy participants had similar gut microbiome composition. Intriguingly, participants with ≥4 seizures in a year have demonstrated a lowered abundance of bacteria belonging to the genera *Bifidobacterium* and *Lactobacillus* [23] (Table 1). On the contrary, the study indicated an increased gut microbial diversity in drug-resistant epilepsy compared to drug-sensitive plus healthy control [23]. Another study carried out by Huang et al. discovered that children with epilepsy and cerebral palsy had a higher microbial diversity, which contradicts the findings by Xie et al. where a lower gut microbial diversity was observed in refractory epileptic infants [22,24] (Table 1). Huang et al. also discovered a higher abundance of *Streptococcus*, *Enterococcus*, *Veillonella*, *Clostridium* IV, *Akkermansia*, *Prevotella*, *Rothia*, and *Bifidobacterium*. Meanwhile, a reduced abundance of *Bacteroides*, *Faecalibacterium*, *Ruminococcus*, *Roseburia*, *Anaerostipes*, *Blautia*, and *Parasutterella* was reported. It is also important to note that epilepsy and cerebral palsy are two different neurological conditions, and there is a possibility of confounding variables affecting the gut microbiota of the patients [24].

**Figure 1:**
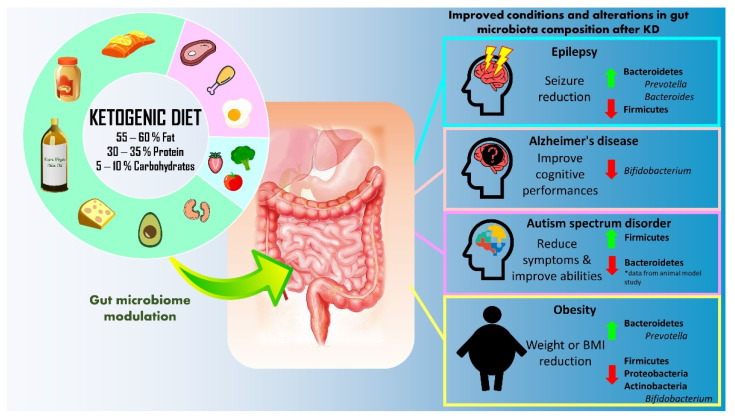
Summary of therapeutic application of ketogenic diet (KD) for epilepsy, Alzheimer’s disease, autism spectrum disorder, and obesity.

In fact, there is limited knowledge on the gut microbiome changes in patients with epilepsy after being treated with KD. Xie and the collaborators reported that 64% of epileptic infants showed a 50% decrease in seizure frequency after one week of KD. The abundance of *Proteobacteria* was lowered dramatically while both *Bacteroidetes* and *Actinobacteria* were increased after KD therapy. However, no differences in *Firmicutes* were noticed. At the genus level, *Bacteroides*, *Prevotella*, and *Bifidobacterium* increased while *Cronobacter*, *Erysipelatoclostridium*, *Streptococcus*, *Alistipes*, *Ruminiclostridium*, *Barnesiella* and *Enterococcus* decreased to a lesser amount [22] (Table 1; Figure 1). Another study involving 20 children with drug-resistant epilepsy suggested that 25% of children achieved ≥50% but <90% of seizure reduction, 15% of children achieved ≥90% of seizure reduction, and 10% of children were seizure-free after 6 months of KD. Their fecal samples after the treatment period were analysed, but there was no significant difference in the alpha diversity. Meanwhile, there was a significant surge in *Bacteroidetes*, but a decline in *Actinobacteria* and *Firmicutes* [25] (Table 1; Figure 1). A recent study was carried out by Lindefeldt et al. (2019) on 12 children with refractory epilepsy treated with KD for three months. The findings displayed that 42% of children had >50% of seizure reduction and 83% of children had improved cognition and motor function. However, their gut microbiota compositions showed no significant difference in terms of alpha diversity. Instead, they exhibited an increase in abundance of *Proteobacteria* (*E. coli*) and a decrease in abundance of *Actinobacteria* (*Bifidobacterium*) [26] (Table 1).

In addition, animal model studies have been conducted to explore the mechanisms behind KD on modulation of the gut microbiome. A mice model experiment demonstrated that KD induced a vital modification in the gut microbiome, mainly causing an increase in abundance of *Akkermansia muciniphila* and *Parabacteroides* spp., with reduced alpha diversity. These modifications of the gut microbiome resulted in changes at the metabolite level, particularly the reduction in gamma-glutamyl amino acid in the colon lumen and blood, accompanied by the rise in the GABA to glutamate ratio in the brain leading to the protection against 6 Hz seizures and reduction in the spontaneous seizure in Kcna1 knockout mice mimicking epilepsy. Furthermore, they depleted the gut microbiota by using mice reared germ-free or providing antibiotics to determine the importance of gut microbiota in affecting the seizure. Consequently, the mice with depleted gut microbiota exhibit decreased seizure threshold even with the introduction of KD. However, the seizure threshold increased when antibiotic-treated mice fed with KD were administered *Akkermansia muciniphila* and *Parabacteroides merdae* [8] (Table 1). In general, the findings of this study suggest that gut microbiota indeed play an important role in seizure protection in addition to KD.

### 2.2. Alzheimer’s Disease

Alzheimer’s disease, a progressive neurodegenerative disease, is the most common type of dementia. The cause is poorly understood, and there is no cure and lack of effective treatment or preventative strategies for this devastating disorder [27,28]. Thus far, Alzheimer’s disease can only be diagnosed clinically and confirmed pathologically by detecting amyloid plaques and neurofibrillary tangles of tau proteins. Research has been revolving around investigating the role of β-amyloid peptides in regard to the development and prevention of this disease [27]. The neuropathological hallmarks of Alzheimer’s disease consist of the accumulation of senile amyloid plaques and neurofibrillary tangles in the brain. β-amyloid peptides are commonly found in plasma and cerebrospinal fluid (CSF), in which a higher percentage of them are identified as amyloid-β 40 (Aβ40) and a small percentage of them are amyloid-β 42 (Aβ42) [29,30]. Aβ42 could aggregate and deposit in the brain, causing the development of senile amyloid plaques in Alzheimer’s patients. As a result, Aβ42 levels are lower in CSF of Alzheimer’s patients [29].

The rising importance of bi-directional communication between the gut microbiome and the brain has prompted researchers to explore the relationship between the gut microbiome and Alzheimer’s disease. The gut microbiome could serve as microbiome biomarkers for Alzheimer’s diagnosis and disease prevention targets [31,32]. In 2017, Vogt et al. discovered a reduced richness based on alpha diversity in the gut microbiome of Alzheimer’s disease patients. Furthermore, there was a decreased abundance in *Firmicutes* and *Actinobacteria* with a concurrently increased abundance of *Bacteroidetes* and *Proteobacteria* (particularly the genus *Bilophila*) in dementia due to Alzheimer’s disease patients as compared to the healthy control participants [33] (Table 1). At the genus level, a higher abundance of genera *Blautia*, *Bacteroides*, *Phascolarctobacterium*, *Alistipes*, *Bilophila*, and *Gamella*, in Alzheimer’s patients were correlated with greater Alzheimer’s pathology through cerebrospinal fluid (CSF) biomarkers. For instance, a lower amyloid-β 42/amyloid-β 40 ratio (Aβ42/Aβ40 ratio) indicates a greater amyloid burden in the brain, a higher phosphorylated tau (p-tau) level indicates a greater tangle pathology in the brain, and a higher phosphorylated tau/amyloid-β 42 ratio (p-tau/Aβ42 ratio) indicates a greater Alzheimer’s disease pathology. A similar trend reflecting greater Alzheimer’s disease pathology was observed when a lower abundance of *Clostridium*, *Bifidobacterium*, *Dialister*, *Turicibacter*, *SMB53*, *Adlercrutzia*, and *cc115* in Alzheimer’s patients. Moreover, Chitinase-3-like protein 1 (YKL-40), a biomarker of Alzheimer’s disease, was found to be increased with a higher abundance of *Bacteroidetes* and a lower abundance of *Firmicutes* (*Turicibacter, SMB53*) [33]. Furthermore, Cattaneo et al. demonstrated an increased pro-inflammatory bacteria (*Escherichia/Shigella*) and a decreased abundance of anti-inflammatory bacteria (*Eubacterium rectale* and *Bacteroides fragilis*) in amyloid-β cognitive impaired elderly’s gut [34] (Table 1).

The gut microbiota composition could potentially contribute to the underlying aetiology of Alzheimer’s disease. Thus, manipulating the gut microbiome composition might influence cerebral amyloid deposition leading to prospective intervention for this disease. Most studies have shown that KD or medium chain triglyceride improves cognitive performance in Alzheimer’s [35,36,37]. In recent years, the reported KD’s benefits include increasing Aβ42 in CSF, decreasing tau protein, and enhancing cerebral perfusion [37]. A clinical trial was conducted to investigate the effect of modified Mediterranean-KD (MMKD) versus American Heart Association Diet (AHAD) on gut microbiota composition in subjects with preclinical stages of Alzheimer’s disease [38]. The study concluded that there was no significant difference in baseline microbiome diversity between participants with or without mild cognitive impairment at risk of Alzheimer’s disease. Nevertheless, patients in the mildly cognitive impaired group had higher levels of *Firmicutes*, *Proteobacteria*, *Tenericutes*, and lower levels of *Bacteroidetes* and *Verrucomicrobia* than that of cognitively normal counterparts. When they probed into the effect on gut microbiota after 6 weeks of MMKD, they discovered that MMKD did no exert significant changes on *Firmicutes*, *Bacteroidetes*, and *Proteobacteria* in both cognitive normal and mild cognitive impaired groups. Nonetheless, there were several changes at the family and genus levels after MMKD, for example, a significant decrease in *Bifidobacterium* (phylum *Actinobacteria*) was clearly observed in mild cognitive impaired group, and this effect was greater than AHAD (Figure 1). In addition to microbial composition, organic fecal acids such as lactate and short-chain fatty acids (SCFAs) were correlated with changes in CSF biomarkers of Alzheimer’s disease. MMKD reduced lactate levels and increased propionate and butyrate SCFAs that could exert positive effects on peripheral and central nervous systems. Above all, the MMKD was associated with decreased gene families annotated to Alzheimer’s disease as predicted by bioinformatics tool [38]. Hence, diet intervention such as KD has the potential to modulate the gut microbiome composition and metabolites that could help improve symptoms of Alzheimer’s disease.

### 2.3. Autism Spectrum Disorder

Autism spectrum disorder is a neurodevelopmental disability affecting children, predominantly in boys rather than girls, and it is recognized by persistent social communication difficulties with repetitive and restrictive behaviors [39]. Several studies have attempted to decipher the connection between the gut microbiome and autism spectrum disorder. Meanwhile, some studies proposed the important role of diet in the clinical manifestation of autism spectrum disorder through modulating the gut microbiome [39,40,41]. According to De Angelis et al., the gut microbiota of autistic children exhibited a higher abundance of *Bacteroidetes* and a lower abundance of *Firmicutes*. At the bacterial genus level, *Clostridium*, *Caloramater* and *Sarcina* were predominantly higher, while *Bifidobacterium* was more deficient in autistic children [42] (Table 1). Moreover, Kandeel et al., 2020 demonstrated the correlation between intestinal *Clostridium* spp. and autism. The study reported increased colonization of these bacteria in autism spectrum disorder children. In particular, *Clostridium difficile* (now reclassified as *Clostridioides difficile*) and *Clostridium clostridioforme* (now reclassified as *Enterocloster clostridioformis*) were only presented in the gut of autistic children, while *Clostridium tertium* only presented in the gut of healthy control children [43]. Therefore, there is a possible correlation between intestinal *Clostridia* colonization and the exacerbation and development of autism spectrum disorder.

The gut microbial diversity of autistic individuals remains elusive, as contradictory reports have presented. De Angelis et al. demonstrated that autistic children had higher microbial diversity in their gut as compared to healthy children [42]. On the contrary, Kang et al. found a less diverse gut microbiome, with a significantly lower abundance of *Prevotella, Coprococcus*, and unclassified *Veillonellaceae* among autistic children. Among these bacteria, *Prevotella* was the dominant genus that differed between neurotypical and autistic children [44]. In one of the latest studies by Ahmed and his colleagues, the alpha diversity was found to be similar in children with autism and healthy control. Moreover, the *Firmicutes*/*Bacteroidetes* (F/B) ratio was significantly lower in autistic children and their non-autistic siblings than in the control group. However, there was no significant result about the respective abundance of *Bacteroidetes* and *Firmicutes*. Moreover, the role of *Prevotella* once again gained its significance when the study reported that *Prevotella* to *Bacteroides* ratio was significantly lower in autistic children [45]. Given these findings, it can be hypothesized that the role of certain gut bacteria overgrowth was one of the factors triggering autism spectrum disorder.

The evidence of KD as a therapeutic approach for autism had been demonstrated in a clinical trial involving 30 children with autism spectrum disorder given KD for 6 months. The outcomes indicated that 7% of participants have significant improvement, while 53% of participants have moderate to minor improvement according to the Childhood Autism Rating Scale. It was noted that milder autism patients achieved the most improvement by KD [46]. El-Rashidy et al. conducted a 6-month case-control study comparing the effect of KD and gluten-free casein-free diet in 45 children with an autism spectrum disorder. Both dietary interventions showed significant enhancement in speech, sociability, cognitive, and behavior, with the improvement in the Childhood Autism Rating Scale and Autism Treatment Evaluation Test. Notably, the KD group exerted better sociability and behavior scores than the gluten-free casein-free group [47].

There is limited research on the effect of KD in modulating the gut microbiome of autistic individuals. Despite that, animal model studies have shown promising evidence that KD alters the growth of certain gut bacterial populations, which may indirectly affect autism symptoms. A murine study by Newell et al. showed that KD increased the F/B ratio in autism spectrum disorder mimicking BTBR mice [48] (Table 1; Figure 1). Following that, the KD also helped to normalize the initially elevated *Akkermansia muciniphila* population in BTBR mice. This bacterium is thought to correlate positively with autism spectrum disorder [42]. It is noteworthy that the total host bacterial abundance was also decreased by 78% in cecum and 28% in feces of BTBR mice after being given KD. This suggested that KD consumption resulted in changes in gut microbiome composition and thus, acknowledged the KD’s promising ability to counteract the common autism spectrum disorder phenotype of low F/B ratio [48]. In another study, BTBR mice fed with KD were proven to improve communication and sociability and reduce self-directed repetitive behavior [49]. Even so, further research is still needed to evaluate the role of KD in altering human gut microbiota composition to confer health benefits in autism patients.

### 2.4. Obesity

KD has been one of the tailored diet regimens to assist in effective weight loss for obese individuals. Obesity is strongly associated with chronic metabolic diseases such as diabetes, hypertension, and increased risk for cardiovascular diseases [50]. The pathogenesis of obesity is due to an energy imbalance, accompanied by other factors, including the composition of our gut microbiome [51,52].

The microbial composition in the gut varied significantly in participants with obesity versus those with normal Body Mass Index (BMI). Meijnikman et al. identified that their gut microbiome alpha diversity was essentially lower among individuals with higher BMI. Approximately 52 bacterial species have differed in the gastrointestinal tract of obese and non-obese individuals. Among the top 10 bacterial taxa identified as predictors of obesity, the obese individuals (BMI > 30 kg m^−2^) possessed a greater abundance of *Actinomyces odontolyticus*, *Streptococcus australis*, *Streptococcus thermophilus*, *Collinsella aerofaciens*, *Granulicatella* spp. and *Lactococcus lactis*. In contrast, individuals without obesity (BMI < 30 kg m^−2^) have a higher abundance of *Alistipes shahii*, *Alistipes senegalensis*, *Lachnospiraceae* sp. 8_157FAA, and *Bacteroidales* sp. ph8. It was proposed that some of these bacteria may play a role in L-histidine biosynthesis, L-lysin biosynthesis, and galactose degradation, for which these metabolic processes were positively correlated with obesity. For instance, the study found that *C. aerofaciens* and *S. thermophilus* were strongly correlated to the histidine biosynthesis pathway. The gut microbiome between individuals with intermediate BMI between 28–35 kg m^−2^ and those with high BMI > 35 kg m^−2^ was also further examined. Results established that the six most predictive species of intestinal microbiota in individuals with severe obesity include *Actinomyces odontolyticus*, *Streptococcus thermophilus*, *Granulicatella* unclassified, *Lactococcus lactis*, and *Collisella aerofaciens*. Overall, the obesity group has a higher abundance of *Firmicutes* (*Ruminococcus torques*, *Ruminococcus obeum*, and *Dorea formicigenerans*) and a diminished abundance of *Bacteroidetes* (*Alistipes shahii* and *Alistipes senegalensis*) [53] (Table 1). Moreover, Turnbaugh et al. revealed an overall reduced gut microbial diversity in obese individuals. A higher abundance of *Actinobacteria* and a lower abundance of *Bacteroidetes* were detected in the gut of obese individuals compared to that of non-obese individuals. The study also implied that gut microbiota could be shared among family members, thus, contributing to the familial pattern of high body weight apart from genetic factors [54]. Schwiertz et al. [55] revealed a lower F/B ratio in obese individuals, as opposed to most findings where a high F/B ratio has been identified in obese individuals [56]. The gut microbiome comprises a complex community that can be affected by various factors such as genetic background, environment, diet, and overall fitness. Henceforth, contradictory findings in gut microbiome-related research were inevitable due to these confounding variables.

Several clinical trials have been conducted to evaluate the therapeutic potential of KD for obesity. A randomized controlled trial involving overweight or obese patients showed that KD was similarly effective compared to a combination of a low-fat diet and lipase inhibitor orlistat in reducing weight [57]. Thus, a high-fat low-carbohydrate diet such as KD can function similarly to calorie intake reduction (low-fat diet + orlistat) in improving obesity. The interrelationship between obesity, hypertension, hyperlipidaemia, and diabetes mellitus could confer elevated risk for cardiovascular diseases [58]. KD significantly reduced blood pressure in obese patients, which showed a more remarkable improvement compared to a low-fat diet + orlistat. As for the lipid profiles, high-density lipoprotein and triglyceride levels were enhanced for patients in both diet groups. Glycaemic parameters, including glucose and haemoglobin A1c levels, were decreased in the KD group [57]. KD poses a promising alternative to treat obesity and metabolic syndrome with the benefits of being relatively simpler and inexpensive than a pharmacological intervention with low-fat dietary intervention.

KD can effectively reduce up to 14% in weight, waist circumference, and BMI, with or without the addition of a symbiotic. This therapeutic dietary plan also modified the gut microbiota by increasing microbial diversity. The proportion of *Proteobacteria* was reduced while the proportion of *Firmicutes* was raised with KD. Furthermore, the bacterial families that decreased in abundance were *Enterobacteriaceae*, *Sinobacteraceae*, and *Comamonadaceae*, whereas those that increased in abundance were *Ruminococcaceae* and *Mogibacteriaceae*. Moreover, the *Bacteroidetes*/*Firmicutes* (B/F) ratio increases along with the higher percentage of weight loss [59] (Table 1). Basciani et al. also concluded that overall *Bacteroidetes* increased in abundance, whilst *Firmicutes* decreased in abundance over 45 days of very-low calorie ketogenic diets (VLCKDs), incorporating whey, vegetable, or animal proteins. When comparing the type of proteins incorporated in KD, the whey or vegetable proteins were more effective in diminishing the abundance of *Firmicutes* than animal protein. Obesity and insulin resistance patients on KD achieved significant weight loss and improved metabolic parameters, including blood pressure, blood glucose, and cholesterol [60] (Table 1). Ang et al. conducted a study on obese (non-diabetics) patients who had shown that after the course of KD, there was a significant increase in abundance of *Bacteroidetes* with decreased abundance of *Firmicutes* and *Actinobacteria* [61] (Table 1). These results are in agreement with the findings that obese individuals had a higher abundance of *Firmicutes* and a lower *Bacteroidetes*, and KD could alter the gut microbiome composition by decreasing the abundance of *Firmicutes* and increasing the abundance of *Bacteroidetes* to restore the balance of the gut ecosystem (Figure 1) [53,61,62].

**Table 1:** The gut microbiome composition in different diseases and the therapeutic effects of KD intervention on disease symptoms and gut microbiome modulation.

Author	Subjects (*n*)	Age (Years Old)	Baseline Gut Microbiome of Patients (Compared to Control Group)	Ketogenic Diet Intervention Period	Key Findings of the Study		Reference
					Clinical Symptoms	Gut Microbiome Alterations	
Epilepsy							
Safak et al. (2020)	Idiopathic focal epilepsy (*n* = 30); Healthy control (*n* = 10)	Adult patients (41.3 ± 12.2); Healthy control (31.7 ± 6.8)	Higher proportion of *Proteobacteria*. Lower proportion of *Firmicutes*. *Fusobacteria* (*Leptotrichia* and *Fusobacterium*) only found in epilepsy group.	N.A. ^1^	N.A.	A significant difference in the gut microbiome composition of idiopathic focal epilepsy patients compared to healthy controls.	[21]
Huang et al. (2019)	Cerebral palsy + Epilepsy children (*n* = 25); Healthy control (*n*= 21)	3 to 18	Higher gut microbial diversity.	N.A.	N.A.	A significant difference in the gut microbiome composition of cerebral palsy and epilepsy children compared to healthy control.	[24]
Peng et al. (2018)	Drug resistant epilepsy (*n* = 42); Drug sensitive epilepsy (*n* = 49); Healthy control (*n* = 65)	Adult patients 28.4 ± 12.4; Adults patients 25.1 ± 14.6; Healthy control 29.4 ± 13.8 years	Higher gut microbial diversity. Higher proportion of *Firmicutes* and *Verrucomicrobia*. Lower proportion of *Bacteroidetes.* Participants with ≥4 seizure in a year have lowered *Bifidobacteria* and *Lactobacillus*.	N.A.	N.A.	A significant difference in the gut microbiome composition of drug resistant epilepsy compared to drug sensitive patients and healthy controls.	[23]
Lindefeldt et al. (2019)	Children with epilepsy (*n* = 12); Healthy parents not starting ketogenic diet as control (*n* = 11)	7.7 ± 4.5	Lower gut microbial diversity.	3 months	5 patients have >50% of seizure reduction (responders); 3 patients had shorter seizures, less postictal tiredness (non-responders); 2 patients did not improve; total 10 patients have improved cognition and motor function.	No significant difference in gut microbial alpha diversity compared to before KD. Increase in *Proteobacteria* (*E. Coli*). Decrease in *Actinobacteria* (*Bifidobacterium*).	[26]
Zhang et al. (2018)	Children with drug resistant epilepsy (*n* = 20)	Children patients 4.2 (range: 1.2 to 10.3)	N.A.	6 months	2 patients with drug resistant epilepsy were seizure free; 3 patients had 90%; 5 patients had ≥50 but less than 90%; 10 patients had <50% decrease in seizure frequency.	No significant difference in gut microbial alpha diversity compared to before KD. Increase in *Bacteroidetes.* Decrease in *Actinobacteria* and *Firmicutes*.	[25]
Xie et al. (2017)	Refractory epilepsy infants (*n* = 14); Healthy control (*n* = 30)	Infant patients 1.95 ± 3.10; Healthy control ≤ 3	Lower gut microbial diversity. Higher proportion of *Proteobacteria* and *Firmicutes*. Lower proportion of *Bacteroidetes* and *Actinobacteria*.	1 week	64% of refractory epilepsy infants showed improvement (21% were seizure free, 43% had 50–90% decrease in seizure frequency).	No significant difference in gut microbial diversity as healthy control. Decrease in *Proteobacteria* Increase in *Bacteroidetes* and *Actinobacteria*.	[22]
Alzheimer’s disease							
Vogt et al. (2017)	Dementia due to Alzheimer’s disease (*n* = 25); Healthy control (*n* = 25)	Adult patients 71.3 ± 7.3; Healthy control 69.3 ± 7.5	Lower gut microbial alpha diversity. Higher proportion of *Bacteroidetes*. Lower proportion of *Firmicutes* and *Actinobacteria*.	N.A.	N.A.	A significant difference in the gut microbiome composition of dementia due to Alzheimer’s disease compared to healthy controls.	[33]
Cattaneo et al. (2017)	Cognitively impaired amyloid-positive patients (Amy+) (*n* = 40); Cognitively impaired amyloid-negative controls (Amy-) (*n* = 33); Cognitively healthy amyloid-negative controls (HC) (*n* = 10)	Adult patients 71 ± 7 years; Adult patients 70 ± 7 years; Healthy controls 68 ± 8 years	Higher proportion of *Escherichia*/*Shigella*. Lower proportion of *Eubacterium rectale* and *Bacillus fragilis*	N.A.	N.A.	A significant difference in the gut microbiome composition of cognitively impaired amyloid-positive patients compared to cognitively impaired amyloid-negative and cognitively healthy amyloid-negative controls.	[34]
Nagpal et al. (2019)	Mild cognitive impaired subject (*n* = 11); cognitive normal subject (*n* = 6)	64.6 ± 6.4	No significant difference in gut microbial diversity. Higher proportion of *Firmicutes*, *Proteobacteria*, and *Tenericutes*. Lower proportion of *Bacteroidetes* and *Verrucomicrobia*.	Modified Mediterrenean-Ketogenic diet (MMKD) for 6 weeks. Note: Only the data related to KD were included for this review purpose	N.A.	No significant difference in gut microbial alpha and beta diversity between two groups after MMKD. No significant difference in abundance of *Firmicutes*, *Bacteroidetes*, and *Proteobacteria* between two groups after MMKD. A significant reduction in abundance of *Bifidobacterium* after MMKD, prominently in mild cognitive impaired participants.	[38]
Autism spectrum disorder							
Kandeel et al. (2020)	Autism spectrum disorder (ASD) (*n* = 30); Neurotypical controls (*n* = 30)	4.4 ± 2.1	Higher proportion of *Clostridium* spp. (*Clostridium paraputrificum* and *Clostridium bolteae*). Two different *Clostridium* spp. (*Clostridium difficile* and *Clostridium clostridioforme*) only found in ASD children. *Clostridium tertium* only found in neurotypical children.	N.A.	N.A.	A significant difference in the microbiome composition of autism children compared to neurotypical controls.	[43]
Ahmed et al. (2020)	Autism Spectrum Disorder (ASD) (*n* = 41); Non autistic sibling group (*n* = 45); Healthy controls (*n* = 45)	Children patients 5.55 ± 1.9 years; Children siblings 4.31 ± 3.23 years; Healthy controls 5.36 ± 2.61 years	No significant difference in gut microbial diversity. Lower proportion of *Firmicutes/Bacteroidetes* (F/B ratio) in both ASD and siblings groups. Lower proportion of *Prevotella* to *Bacteroides* ratio (P/B ratio) in both ASD and siblings groups.	N.A.	N.A.	A significant difference in the gut microbiome composition between groups.	[45]
De Angelis et al. (2013)	Pervasive Developmental Disorder Not Otherwise Specified (PDD-NOS) (*n* = 10); Autism (AD) (*n* = 10) Healthy controls (HC) (*n* = 10)	4 to 10	Higher gut microbial diversity in autism children. Higher proportion of *Bacteroidetes*. Lower proportion of *Firmicutes*.	N.A.	N.A.	A significant difference in the gut microbiome composition of autism children compared to PDD-NOS and healthy controls.	[42]
Kang et al. (2013)	Autism Spectrum Disorder (*n* = 20); Neurotypical controls (*n* = 20)	Children patients 6.7 ± 2.7; Healthy controls 8.3 ± 4.4	Lower gut microbial alpha diversity (Phylogenetic Diversity index). Lower proportion of *Prevotella*, *Coprococcus,* and *Veillonellaceae*.	N.A.	N.A.	A significant difference in the microbiome composition of autism children compared to neurotypical controls.	[44]
Obesity							
Meijnikman et al. (2020)	Obesity (*n* = 95); Non-obesity control (*n* = 82)	Adult patients 47.1 ± 10.8; Non-obesity control 24.7 ± 2.9	Lower gut microbial diversity in obese individuals. Top 10 bacterial taxa as predictors of obesity: Higher abundance in obese individuals (BMI > 30 kg m^−2^) *1.* *Actinomyces odontolyticus* *2.* *Streptococcus australis* *3.* *Streptococcus thermophilus* *4.* *Collinsella aerofaciens*, *5.* * Granulicatella* spp. *6.* *Lactococcus lactis* Higher abundance in non-obese individuals (BMI < 30 kg m^−2^) *7.* *Alistipes shahii* *8.* * Alistipes senegalensis* *9.* *Lachnospiraceae* sp. 8_157FAA *10.* *Bacteroidales* sp. ph8. Overall, higher proportion of *Firmicutes* and lower proportion of *Bacteroidetes* in obese individuals.	N.A.	N.A.	A significant difference in the gut microbiome composition of obese individuals compared to non-obese control.	[53]
Ang et al. (2020)	Over-weight / class I obese non-diabetic men (*n* = 17)	Adult patients	N.A.	Baseline diet (4 weeks) followed by KD (4 weeks)	N.A.	Increase in abundance of *Bacteroidetes*. Decrease in abundance of *Actinobacteria*, *Firmicutes*. Greatest decrease in abundance of *Bifidobacterium*.	[61]
Basciani et al. (2020)	Obese patients (*n* = 48)	Adult patients 56.2 ± 6.1	N.A.	Very-low-calorie ketogenic diets (VLCKDs), incorporated whey protein, plant protein, and animal protein (for 45 days).	Obese and insulin resistance patients in all KD groups showed reduction in BMI, body weight, waist circumference, blood pressure, HOMA index, insulin, and total LDL cholesterol.	No significant difference in the gut microbiome composition with different types of protein. Increase in *Bacteroidetes*. Decrease in *Firmicutes*.	[60]
Gutiérrez-Repiso et al. (2019)	Obese patients (*n* = 33)	Adult patients 48.67 ± 9.16 years; Adult patients 47.00 ± 8.97 years; Adult patients 38.22 ± 11.27 years	N.A.	Very low calory KD and symbiotic (*Bifidobacterium animalis* subsp. *Lactis* and prebiotics fiber), 3 groups: Synbiotic1 + Synbiotic2; Placebo + Synbiotic2; Placebo only (4 months)	KD (placebo + Synbiotic 2) showed a significantly highest percentage of weight loss, −14.10 ± 3.89 (%). Overall, KD caused a reduction in weight, waist circumference, and BMI.	Symbiotic did not affect gut microbial diversity, but increased the abundance of *Odoribacter* and *Lachnospira*. KD increased gut microbial diversity. Decrease in abundance of *Proteobacteria*. Increase in abundance of *Firmicutes*. *Bacteroidetes/Firmicutes* ratio increases with the higher percentage of weight loss after KD.	[59]
Turnbaugh, et al., 2009	Participants (*n* = 154)	Adults between 21–32 years	Lower gut microbial diversity in obesity participants. Higher proportion of *Actinobacteria*. Lower proportion of *Bacteroidetes*.			Significant differences in the gut microbiome composition of obesity patients compared to non-obesity controls.	[54]

^1^ N.A.: Not available.

## 3. Discussion

The utilization of KD as a therapeutic regime has grown in popularity for various diseases, including epilepsy, Alzheimer’s disease, autism spectrum disorder, and obesity. To date, there are no standard guidelines regarding application of KD for physical and mental illnesses. Nonetheless, Kraeuter et al. suggested the best practices of KD for implementation in preclinical and clinical studies [63]. The growing significance of the gut microbiome in the pathogenesis of these diseases has implicated that manipulating gut microbiota through dietary intervention such as KD could provide unprecedented opportunities to attenuate, treat or prevent these diseases in the near future [64,65,66].

Epileptic and Alzheimer’s patients have shown a higher proportion of intestinal *Proteobacteria*. *Proteobacteria* comprising pathobionts have been reported to favor inflammation and resulted in colorectal cancer and autoimmune diseases such as inflammatory bowel diseases [67,68]. As the gut microbiome could contribute to neurological disease through the gut–brain axis, *Proteobacteria* could drive neurological inflammation, leading to epilepsy and Alzheimer’s disease. For example, the reduction of intestinal *Proteobacteria* by KD can be inferred from the study by Xie et al., which ultimately led to a 50% decrease in seizure frequency among 9 out of 14 infants with refractory epilepsy [22]. Nonetheless, evidence on the effectiveness of KD in altering the *Proteobacteria* population remains insufficient to offer an indisputable conclusion.

Generally, epileptic patients have shown a lower abundance of *Bacteroidetes* in the gut, and the abundance of these bacteria increases after a period of KD consumption. For instance, the genus *Bacteroides* (family *Bacteroidetes*) was accumulated in infants with epilepsy after KD treatment. *Bacteroides* genus consists of a group of bacteria capable of regulating the release of inflammatory cytokines such as Interleukin 6 (IL-6) and Interleukin 17 (IL-17) in dendritic cells, which are highly correlated with seizure severity [22,69]. The increase in B/F ratio is associated with increased production of short-chain fatty acid, which corresponds with a short-chain fatty acid antiepileptic drug known as valproic acid [70,71]. Basciani et al. [60] also discovered that *Bacteroidetes* increased while *Firmicutes* decreased over 45 days of KD. Likewise, Gutiérrez-Repiso et al. [59] concluded that a rise in B/F ratio is associated with a higher weight loss percentage. The increased abundance of *Bacteroidetes* and decreased abundance of *Firmicutes* were similarly observed across 17 non-diabetic obese adults after KD [61]. The increased production of short-chain fatty acid could modulate peptide tyrosine-tyrosine (PYY) and glucagon-like peptide-1 (GLP-1), inducing satiety and reducing the intake of food. GLP-1 is essential in mediating insulin in the body [72]. Thus, an increased B/F ratio could benefit obesity and type 2 diabetes mellitus. Nevertheless, the only trial probed into the manipulation of gut microbiota by KD in Alzheimer’s disease did not show any significant difference in the B/F ratio [38]. In contrast, a lower F/B ratio was observed in Alzheimer’s disease patients and autistic children. In other words, a higher B/F ratio was instead associated with autism spectrum disorder and Alzheimer’s disease [33,38,45]. Findings on the association of F/B ratio with these diseases were inconsistent. Hence, the significance of F/B ratio as a disease marker remains inconclusive.

Apart from that, bacteria at the genus level, particularly *Prevotella*, may play an important part in promoting gut health. The importance of *Prevotella* as a healthy-gut biomarker could be viewed in the increase in abundance of *Prevotella* in infants with refractory epilepsy after being treated with KD [22]. The abundance of *Prevotella* also increased significantly in obese adults after KD [61]. Moreover, autism patients have a lower level of *Prevotella* [44]. *Prevotella* sp. is one of the producers of vitamin B1 (thiamine) [73]. Vitamin B1 deficiency has been previously detected in autistic children and identified as a risk factor for autism spectrum disorder [74]. Thus, the lack of these bacteria in autism patients could be associated with the development of their symptoms. The effect of KD on *Prevotella* population in the gut of autism patients is yet to be determined. Nevertheless, their growth can be promoted by fish oil supplementation, which is vital for optimal brain development and attenuating symptoms of autism spectrum disorder, given their role in vitamin B1 synthesis [44].

Another essential gut microbiota is the genus *Bifidobacterium*. This group of bacteria is often associated with various therapeutic properties towards many diseases such as colorectal cancer, diarrhea, necrotizing enterocolitis, and inflammatory bowel disease [75]. *Bifidobacterium* could promote the synthesis of gamma aminobutyric acid, an inhibitory neurotransmitter to suppress seizures [23,76]. However, several studies compiled in this review had reported that *Bifidobacterium* in the gut was depleted after KD [26,38,59]. A study conducted by Olson et al., 2020 revealed that the ketone bodies produced upon KD had selectively inhibited the growth of *Bifidobacterium*, subsequently reducing their population in the gut [61]. *Bifidobacterium* (for instance, *Bifidobacterium adolescentis*) has a role in inducing intestinal Th17 cells—a class of pro-inflammatory immune cells. Hence, the decreased abundance of *Bifidobacterium* could reduce the levels of pro-inflammatory Th17 cells [61,77]. As a result, the modulation of pro-inflammatory Th17 cells through KD can be a promising therapeutic approach to alleviate immunological, neurological, and metabolic disease symptoms.

Previous studies have shown the beneficial effects of KD on alleviating symptoms of epilepsy, Alzheimer’s disease, autism spectrum disorder, and obesity. However, the findings on KD’s impact on gut microbiome alteration remain scarce. Future research on the therapeutic application of KD should incorporate the component of gut microbiome analysis. This can further enhance the understanding of the connection between diet, gut microbiome, and diseases. Moreover, clinical trials have proven that the gut microbiome varied after KD, but some outcomes indicated that the microbial changes were inconsistent across different studies. The reasons behind this could be the small sample size and the short duration of implementation of KD. Therefore, future study with larger sample sizes and extended KD periods is crucial to examine long-term safety and generate more reliable results on the impact on gut microbiota. The lack of capacity in children and the elderly with neurological conditions might be an additional challenge. In addition, the implementation of KD might be challenging due to increased food selectivity and difficulties in communication and behavioral flexibility, especially with autistic children [78].

Furthermore, it is crucial to address the potential adverse effects of consuming a KD. For example, the risk of gastrointestinal effects (constipation, diarrhea, vomiting, pancreatitis, hepatitis), electrolyte imbalances (hypomagnesemia, hyponatremia), metabolic dysfunction (hyperuricemia, transient hyperlipidemia) [79,80,81]. Prolonged effects include vitamin deficiency, osteopenia, neurological dysfunction, atherosclerosis, hepatotoxicity, nephrolithiasis, and anemia. Impaired sense of smell and taste, early satiety, dysphagia have been reported in patients with neurocognitive disorder after consuming KD [80]. The adjustment in food intake might trigger other health complications since these populations were at higher risk for malnutrition. Hence, it is necessary to examine the safety profile and consult a licensed nutritionist before implementing KD as a therapeutic diet [63].

## 4. Conclusions

The gut microbiome composition has been proposed to be one of the factors contributing to the pathogenesis of neurological and nutritional diseases, such as epilepsy, Alzheimer’s disease, autism spectrum disorder, and obesity. As diet is a vital modifiable factor that regulates gut microbiome composition, implementing KD could produce therapeutic outcomes. Studies have demonstrated that KD could confer health benefits to individuals with epilepsy, Alzheimer’s disease, autism spectrum disorder, or obesity. KD alleviated the disease symptoms, and this could be associated with the alteration of the gut microbiome. KD potentially decreases the intestinal colonization of *Proteobacteria* that associated with pro-inflammatory responses. Additionally, KD increases B/F ratio which particularly benefits obese individuals and increases the levels of other beneficial intestinal microbes such as *Prevotella*. However, there are still insufficient studies to deduce the effect of KD on gut microbiome modulation, and more evidence is required to evaluate its effectiveness as a therapeutic diet. The inconsistency in the changes of gut microbial composition and diversity by KD also warrants further investigation. Future studies with a larger sample size and longer KD courses are recommended to examine the effects of KD on gut microbiome modulation and the associated therapeutic implications. It is also critical to monitor the long-term beneficial effects and potential adverse events of KD.

## Data Availability

Not applicable.
